# Mitochondrial DNA Variation and Introgression in Siberian Taimen *Hucho taimen*

**DOI:** 10.1371/journal.pone.0071147

**Published:** 2013-08-12

**Authors:** Evgeniy S. Balakirev, Nikolai S. Romanov, Pavel B. Mikheev, Francisco J. Ayala

**Affiliations:** 1 Department of Ecology and Evolutionary Biology, University of California Irvine, Irvine, California, United States of America; 2 A. V. Zhirmunsky Institute of Marine Biology, Far Eastern Branch, Russian Academy of Science, Vladivostok, Russia; 3 Amur River Salmon Sector, Pacific Research Fisheries Centre (TINRO-Centre), Khabarovsk Branch, Khabarovsk, Russia; CSIRO, Australia

## Abstract

Siberian taimen *Hucho taimen* is the largest representative of the family Salmonidae inhabiting rivers of northern Eurasia. The species is under intensive aquaculture activity. To monitor natural taimen populations we have sequenced a portion (8,141 bp) of the mitochondrial (mt) genome in 28 specimens of *H. taimen* from six localities in the Amur River basin. Nucleotide variability is low (π = 0.0010), but structured in two divergent haplotype groups. A comparison of the data with the GenBank *H. taimen* mt genome (HQ897271) reveals significant differences between them in spite of the fact that the fish specimens come from neighboring geographical areas. The distribution of divergence is non-uniform with two highly pronounced divergent regions centered on two genes, *ND3* and *ND6*. To clarify the pattern of divergence we sequenced the corresponding portion of the mt genome of lenok *Brachymystax tumensis* and analyzed the GenBank complete mt genomes of related species. We have found that the first and second divergent regions are identical between the GenBank *H. taimen* and two lenok subspecies, *B. lenok* and *B. lenok tsinlingensis*, respectively. Consequently, both divergent regions represent introgressed mtDNA resulting from intergeneric hybridization between the two lenok subspecies and *H. taimen*. Introgression is, however, not detected in our specimens. This plus the precise identity of the introgressed fragments between the donor and the recipient GenBank sequence suggests that the introgression is local and very recent, probably due to artificial manipulations involving taimen – lenok intergeneric hybridization. Human-mediated hybridization may become a major threat to aquatic biodiversity. Consequently we suggest that due attention needs to be given to this threat by means of responsible breeding program management, so as to prevent a potential spread of hybrid fishes that could jeopardize the resilience of locally adapted gene pools of the native *H. taimen* populations.

## Introduction

Siberian taimen *Hucho taimen* (Pallas, 1773) is the world’s largest salmonid fish, reaching up to 2 m in length and 105 kg in weight [1]–[3]. The maximum size and weight of the species are less in the Amur River: 1.5 m and 10 kg (up to 80 kg), respectively [4]. The long life span (20–29 years) and relatively high age at maturity (7–9 years) indicate that the species is vulnerable to overfishing. Indeed, Siberian taimen has significantly declined in abundance throughout their range in the large rivers of the Volga/Caspian, Arctic, and Pacific drainages of northern Eurasia and it is now included as vulnerable species in the Mongolian, Russian, and Chinese red lists [5]–[7].

The unique biological features and severe decline of taimen populations have stimulated intensive genetic investigations of the species. Intraspecific genetic variation in Siberian taimen has been investigated by means of chromosome analysis [8], allozymes [9], microsatellites [10]–[13], AFLP markers [14]–[15], and DNA sequences [16]–[18]. The level of intraspecific variability detected is generally low and correlated with an increase in habitat degradation and excessive fishing [12], [15]. Significant population differentiation has been found at a large geographic scale, extending through the main Siberian river basins [16]–[17], as well as at a much smaller geographic scale, between different tributaries within the Amur (Heilongjiang) River basin [12], [13], [15]. Significant genetic differentiation along with low nucleotide sequence diversity have also been found in the European huchen, *H. hucho* (Linnaeus, 1758) [19]–[20]. This population structure might be explained by the relatively low dispersion capability of the fish and/or the homing like behavior suggested for *H. hucho* [1]. In *H. taimen*, individual home range, obtained with radio and acoustic telemetry, is 0.5–93.2 km (mean ± SD, 23±25.7 km) [21]. Anthropogenic factors may also reduce gene flow between taimen populations [12], [15].

The aquaculture of *H. taimen* is under intensive development; it includes artificial rearing and reproduction [22]–[24], hybridization [25]–[28], and propagation [29]. Artificial intergeneric hybrids between *H. taimen* and lenok *Brachymystax lenok* (Pallas, 1773) (which represent the most recent split (∼19.9 MY) between Salmonidae genera; [30]) have been reported [25]–[27]. Heterosis was observed for the hybridization between *H. taimen* ♀ and *B. lenok* ♂ [26]; however the opposite parental combination (*H. taimen* ♂ and *B. lenok* ♀) yielded hybrid incompatibility [27].

*H. taimen* and *B. lenok* are sympatric in the Amur River drainages [4], [31] but natural hybrids between them are very rare (our own personal observations as well as communications from Dr. Antonov A.L., Institute of Water and Ecological Problems, Far Eastern Branch, Russian Academy of Sciences, Khabarovsk, Russia; and Dr. Ma B., Heilongjiang River Fisheries Research Institute, Chinese Academy of Fishery Sciences, Harbin, China). Natural hybrids between taimen and lenok have, however, been described in Chinese literature [32]. Using mitochondrial DNA (mtDNA) restriction fragment length polymorphism analysis, Shedko [33]–[34] detected alien haplotypes in lenok, supposedly due to hybridization between lenok and taimen. Indeed, one of the studied lenok specimens had the mtDNA haplotype typical for taimen, but morphologically showed intermediate characteristics between lenok and taimen, suggesting a possible hybridization event between lenok and taimen. Rare hybrid individuals have been detected only in the Khanka Lake basin [33]–[34]. This area is characterized by relatively high human population density, so that human-mediated hybridization is possible. Hybrid individuals were not detected in any other Primorye Territory sampling points where lenok and taimen occur in sympatry [33]–[34]. It has been shown that females of taimen *H. taimen*, different to most other salmonid fishes, do not cover their eggs immediately after having spawned, but rest for a variable number of minutes before covering them [35]. They share this unusual trait with females of lenok *B. lenok* [36]. Thus, the female reproductive (spawning) behaviors of lenok *B. lenok* and taimen *H. taimen* have some similarities which suggests that breeding between these species is theoretically possible.

The *H. taimen* – *B. lenok* putative hybrid individuals were characterized by mtDNA restriction fragment length polymorphism analysis [33]–[34], sequence – related amplified polymorphisms [26], and microsatellite markers [27]. However, direct evidence of hybridization based on the nucleotide sequences was not obtained. Among the different types of nucleotide sequence markers, mtDNA is particularly appropriate to reveal and describe possible introgression in hybrid individuals (e.g., [37]–[38]). Indeed, several studies of gene flow between closely related species have found that some parts of the genome introgress more easily than others. For example, mtDNA tends to introgress more readily than nuclear DNA (nuDNA) (e.g., [39]–[42]). It has been suggested that mtDNA may not include loci that contribute to hybrid unfitness or positive assortative mating, and that most mitochondrial genes, unlike nuclear genes, may function fairly well in the genetic background of a related species [43]; see [41]–[42] for further potential explanations for stronger signatures of introgression of mtDNA compared to nuDNA.

Hybridization and propagation are common in *H. taimen* aquaculture practice [22]–[29]. Consequently, negative genetic effects are expected (see Discussion) and should be under control to prevent deterioration of natural taimen populations. In this study, we have sequenced a fragment of the mt genome (8,141 bp) in 28 individuals of *H. taimen* from six localities of the Amur River basin to test whether *B. lenok* alleles have introgressed into populations of *H. taimen*. We found all specimens collected free from introgression. However, the mtDNA of the *H. taimen* specimen (GenBank HQ897271) from the Hutou range (Ussuri River, China) clearly indicates contemporary introgression due to hybridization with lenok subspecies, *B. lenok* and *B. lenok tsinlingensis* Li, 1966. We conclude that genetic monitoring presents an important and reliable approach to identify hybrid individuals and to assess the threat from hybridization to the genetic integrity of *H. taimen* natural populations.

## Materials and Methods

### Fish Samples

The 28 specimens of *H. taimen* were collected from six rivers: Nora, Bikin, Manoma, Anyuy, Sutara, and Khor, all from the Amur River basin. A single specimen of blunt-snouted lenok *Brachymystax tumensis* Mori, 1930 was collected from the Bikin River. Sampling locations of *H. taimen* are shown in Fig. S1. The river names, basins, sample sizes, and coordinates are presented in Tables S1 and S2. Additionally we used full mt genomes from GenBank: 1) *H. taimen* (HQ897271), 2) *H. bleekeri* Kimura, 1934 (HM804473), 3) *B. lenok* (JQ686730), and 4) *B. lenok tsinlingensis* (JQ675732, JQ686731). The nucleotide sequences of *Salmo salmo* Valenciennes, 1848 (*S. salmo* Valenciennes, 1848 is a junior synonym of *S. salar* Linnaeus, 1758) (U12143, AF133701), *S. trutta* Linnaeus, 1758 (AM910409), *Salvelinus fontinalis* (Mitchill, 1814) (AF154850), and *S. alpinus* Linnaeus, 1758 (AF154851) were used as outgroups. Full mt genomes of related salmonids, included in the present analysis as outgroup taxa, were selected based on previous molecular evidence of close relationship to *H. taimen* [30] and screening of nucleotide sequences available in GenBank.

### Ethics Statement

*H. taimen* and *B. tumensis* are listed as “vulnerable” species in the Red Data Book of some constituent territories of the Russian Federation. However, they are considered as “commercial” species in the Amur Region, Primorye Territory, Khabarovsk Territory, and Jewish Autonomous Region, where we collected the specimens for the present study. Limited fishery is officially permitted in these areas. The described field studies were based on the quota limit obtained from the Federal Agency for Fishery of the Russian Federation (order #1172, November 28, 2011; signed by the FAF Deputy Director, V. I. Sokolov; see details, http://www.garant.ru/products/ipo/prime/doc/70002838/). The sampling points are located beyond the protected territories of the Amur River basin (see map and details, http://amur-heilong.net/http/04_econet_pas/0405RussiaPAs.html). The field studies did not involve endangered or protected species. The locations of the field studies are not privately-owned or protected. The fishes were collected with non-lethal gear and sacrificed after anaesthetization using a solution (75–200 mg/l) of tricaine methanesulfonate (ethyl 3-aminobenzoate methanesulfonate, MS-222; Sigma-Aldrich, E10521) or clove oil (eugenol; Sigma-Aldrich, W246719). The present field study was approved by the Federal Agency for Fishery of the Russian Federation, which has the highest decision authority concerning fish care and use and should be considered as an equivalent to the Institutional Animal Care and Use Committee.

### DNA Amplification, Cloning, and Sequences

Total genomic DNA was extracted using the DNeasy Blood and Tissue kit (Qiagen, Hilden, Germany) from 96% ethanol-preserved muscle tissue of *H. taimen*. The procedures for DNA amplification, cloning, and sequencing have been described previously [44]–[45]. The mtDNA fragment (8.1 kb) was amplified with primers designed with the program mitoPrimer, version 1 [46]. The primers are listed in Table S3. The region of *H. taimen* mt genome, with the corresponding coordinates of mt genomes of *H. taimen* [47], *H. bleekeri* [48] and *B. lenok tsinlingensis* [49] are given in Table S4.

The PCR reactions were carried out in final volumes of 25 µl using TaKaRa Ex Taq^™^ in accordance with the manufacturer’s description (Takara Biotechnology Co., Ltd.). The PCR reaction mixtures were placed in a DNA thermal cycler (Eppendorf, Mastercycler Gradient), incubated 5 min at 95° and subjected to 32 cycles of denaturation, annealing, and extension: 94° for 30 sec, 54°–60° (depending on the Tm of the particular primer pair, see Table S3) for 30 sec, and 72° for 1.0 min, with a final 7-min extension period at 72°. The PCR products were sequenced by the dideoxy chain-termination technique using Dye Terminator chemistry and separated with the ABI PRISM 377 automated DNA sequencer (Perkin Elmer). The sequences of both strands were determined, using overlapping internal primers spaced 500 nucleotides, on average. At least two independent PCR amplifications were sequenced in both directions to correct for possible errors. The heteroplasmic region was resolved by cloning using the Topo-TA cloning chemistry (Invitrogen). The sequences were annotated with the program DOGMA [50] and deposited in GenBank under accession numbers KC920394–KC920422, KC936238, and KC936240–KC936267.

### DNA Sequence Analysis

The sequences were assembled using the program SeqMan (Lasergene, DNASTAR, Inc., version 10.1). Multiple alignments were carried using the program CLUSTAL W [51]; overlapping regions (*ND4L* – *ND4*, *ND5*– *ND6*; see Table S4) were excluded from the analysis. The computer programs DnaSP, version 5 [52] and PROSEQ, version 2.9 [53] were used for most intraspecific analyses.

Model-based phylogeny reconstructions were performed with concatenated sequence alignments using the neighbor-joining (NJ), maximum-likelihood (ML), and Bayesian algorithms, using respectively the programs MEGA, version 5 [54], GARLI 2.0 [55], and MrBayes 3.2 [56]. For all reconstructions, the best-fit model of nucleotide substitution was chosen with the Akaike Information Criterion (AIC) and the Bayesian information criterion (BIC) in MEGA and jModelTest, version 2 [57] (Table S5). Maximum likelihood bootstrap analyses [58] consisted of 1000 replicates. In ML analysis, only the model specification settings were adjusted according to the respective concatenated dataset, while all other GARLI settings were left at their default value. In Bayesian inference, Markov Chain Monte Carlo (MCMC) was run under a general-time-reversible model plus gamma plus I (GTR+G+I). Analyses were performed as four independent MCMC runs each with three incrementally heated chains and a single cold chain for two million generations. Output trees and data were sampled every 500 generations. Likelihood values reached a plateau within approximately 1,000 generations. Omitting the first 5,000 (25%) burn-in trees, the remaining trees were used to estimate the 50% majority rule consensus trees and the Bayesian posterior probabilities. At the end of the run there were not any trends in a plot of generation versus the log probability of the observed data (the log likelihood values). A convergence diagnostic, the Potential Scale Reduction Factor (PSRF) [59] was between 1.000 and 1.003 indicating a good sample from the posterior probability distribution. All MCMC runs were repeated to confirm consistent approximation of the posterior parameter distributions.

Partitioned analyses were performed with GARLI, which allowed the overall rate to be different across each separate mtDNA protein-coding gene included in the present analysis. The partitioned dataset treats each locus separately and each with its own substitution model, while the unpartitioned dataset was regarded as one partition. Unpartitioned data sets included the entire 8,141 bp concatenated dataset (Table S4) and the protein-coding genes only (7,608 bp without stop-codons). Partitioned analyses included (1) each protein-coding gene as a separate partition, (2) the same as (1) but excluding *ND3*+ *ND6*, (3) *ND3* only, (4) *ND6* only, and (5) *ND3*+ *ND6* only (see Results). The topologies obtained with the NJ and ML methods, as well as, by Bayesian inference, were congruent and received high support in most nodes. To be conservative we show the lowest bootstrap values obtained with the ML method.

### Recombination Analysis

The alignments were analyzed for evidence of recombination using various recombination detection methods implemented in the program RDP3 [60]. The parental and recombinant sequences were determined using the VisRD method [61], modified version of PHYLPRO [62], and EEEP [63] also implemented in RDP3 (default settings).

## Results

### Nucleotide Diversity and Divergence

**Figure 1:**
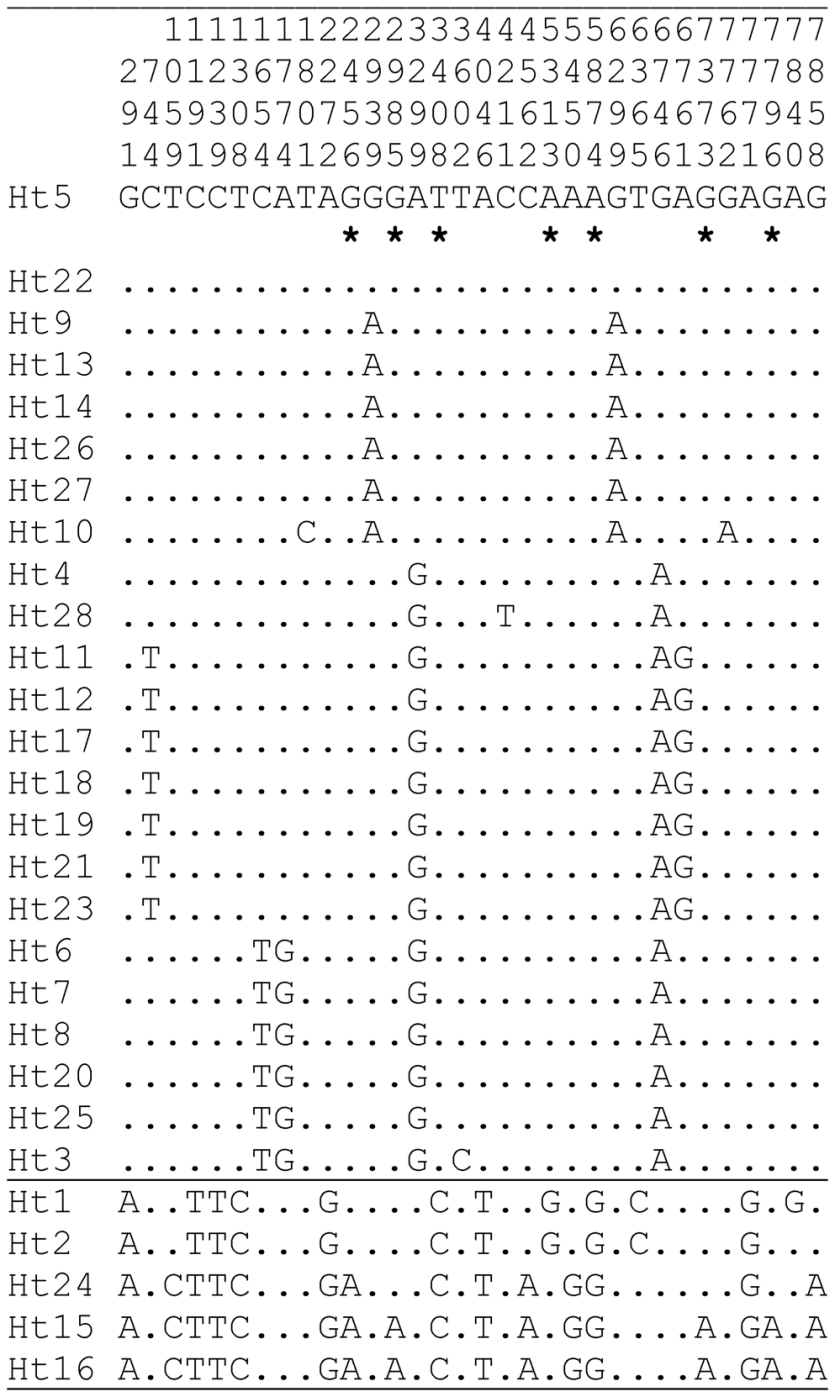
Nucleotide polymorphism in the mtDNA of *Hucho taimen*. Numbers above the top sequence give the position of segregating sites. The samples are listed sequentially according to their genetic similarity. Nucleotides are numbered from the beginning of our sequence, position 6,663 in the complete mitochondrial genome of *H. taimen* [47]. Amino acid replacement polymorphisms are marked with asterisks below the reference sequence (Ht5). Dots indicate the same nucleotide as the reference sequence. The coordinates for the functional regions are in Table S4. The figure presents the polymorphic sites revealed in the full mtDNA fragment sequenced (8,141 bp), including coding and non-coding regions in all *H. taimen* specimens (see Fig. S1 and Table S1 for the collection sites and specimen designation).

We sequenced a 8,141 bp fragment of mitochondrial genome in 28 *H. taimen* specimens. The fragment included eight protein-coding genes: *COI*, *COIII*, *ND3*, *ND4L*, *ND4*, *ND5*, *ND6*, and *CYTB*, and eight transfer RNAs genes (see Table S4 for coordinates). Fig. 1 shows 32 nucleotide substitution sites (5 singletons and 27 informative sites) detected in the 28 *H. taimen* specimens (6 sites in *COI*, 3 sites in *COIII*, 2 sites in *ND3*, 7 sites in *ND4*, 5 sites in *ND5*, 3 sites in *ND6*, and 6 sites in *CYTB*). No polymorphic sites were detected in *ND4L* or in the transfer RNAs genes. No length polymorphisms were found. Total nucleotide diversity was low (π = 0.0010); the results are in accordance with data on intraspecific variability in *H. taimen* [16]–[17] and close species, *H. hucho* [20] and *H. bleekeri* [64].

There were 25 synonymous and 7 replacement polymorphic sites in the coding regions of the eight genes studied (7,608 bp; 2,536 codons totally). Replacement substitutions were detected in *ND3* (1 site), *ND4* (2 sites), *ND5* (2 sites), and *CYTB* (2 sites). All replacements were non-singletons (Fig. 1). Total nucleotide diversity in the coding region was π = 0.0011. Synonymous variability (π = 0.0035) was 17.5 times higher than nonsynonymous variability (π = 0.0002), indicating a high level of negative selection on amino acid substitutions in the mtDNA of Siberian taimen.

Strong haplotype structure was detected with two main haplotype groups. The first and second group include 23 and 5 sequences, respectively (separated by a horizontal line in Fig. 1) with 9 fixed single nucleotide differences between them. The genetic structure is completely congruent for all gene regions studied. The second haplotype group was associated with the replacement polymorphism at positions 2456, 2985, 3408, 5313, 5874, 7373, and 7796 (Fig. 1). The difference between the groups based on the full concatenate (8,141 bp) is highly significant (*F*_st_ = 0.7059, *P*<0.0001); total sequence divergence (*D*_xy_) between the groups is 0.0021±0.0005. Both haplotype groups, however, can be present within a single locality (Table S1) and have similar values of nucleotide diversity estimates (Table 1).

**Table 1:** Nucleotide diversity and divergence in the protein coding mitochondrial genes of *Hucho taimen* for the haplotype group 1 (A), haplotype group 2 (B), and full sample (C).

	N	S	π	θ	h	*K_tai-taiH_*	*K_tai-ble_*	*K_tai-len_*	*K_tai-len-t_*
A									
Syn	1915	12 (4)	0.0020	0.0017		0.0413	0.0750	0.3055	0.3114
Nsyn	5693	0 (0)	0	0		0.0011	0.0026	0.0047	0.0054
Total	7608	12 (4)	0.0005	0.0004	8	0.0110	0.0202	0.0699	0.0715
B									
Syn	1916	6 (1)	0.0018	0.0015		0.0468	0.0755	0.3085	0.3133
Nsyn	5692	5 (0)	0.0005	0.0004		0.0018	0.0030	0.0055	0.0060
Total	7608	11 (1)	0.0008	0.0007	4	0.0129	0.0206	0.0711	0.0723
C									
Syn	1915	25 (5)	0.0035	0.0034		0.0423	0.0751	0.3060	0.3118
Nsyn	5693	7 (0)	0.0002	0.0003		0.0012	0.0027	0.0049	0.0055
Total	7608	32 (5)	0.0011	0.0011	12	0.0113	0.0203	0.0701	0.0716

Note. – N, number of sites; S, number of polymorphic sites (singletons are in parentheses); π, average number of nucleotide differences per site among all pairs of sequences [65] obtained for the synonymous (Syn), nonsynonymous (Nsyn), and total number of sites; θ, average number of segregating nucleotide sites among all sequences, based on the expected distribution of neutral variants in a panmictic population at equilibrium [66]; h, number of haplotypes; *K_tai-taiH_*, *K_tai-ble_*, *K_tai-len_*, and *K_tai-len-t_* refer to the average proportion of nucleotide differences between our specimens of *H. taimen* and the GenBank *H. taimen*, *H. bleekeri*, *B. lenok* or *B. lenok tsinlingensis*, respectively. The *H. taimen* GenBank specimen from the Hutou range of the Ussuri River in Heilongjiang Province (GenBank HQ897271) is not considered in this analysis because it includes introgression events (see Results).

**Figure 2:**
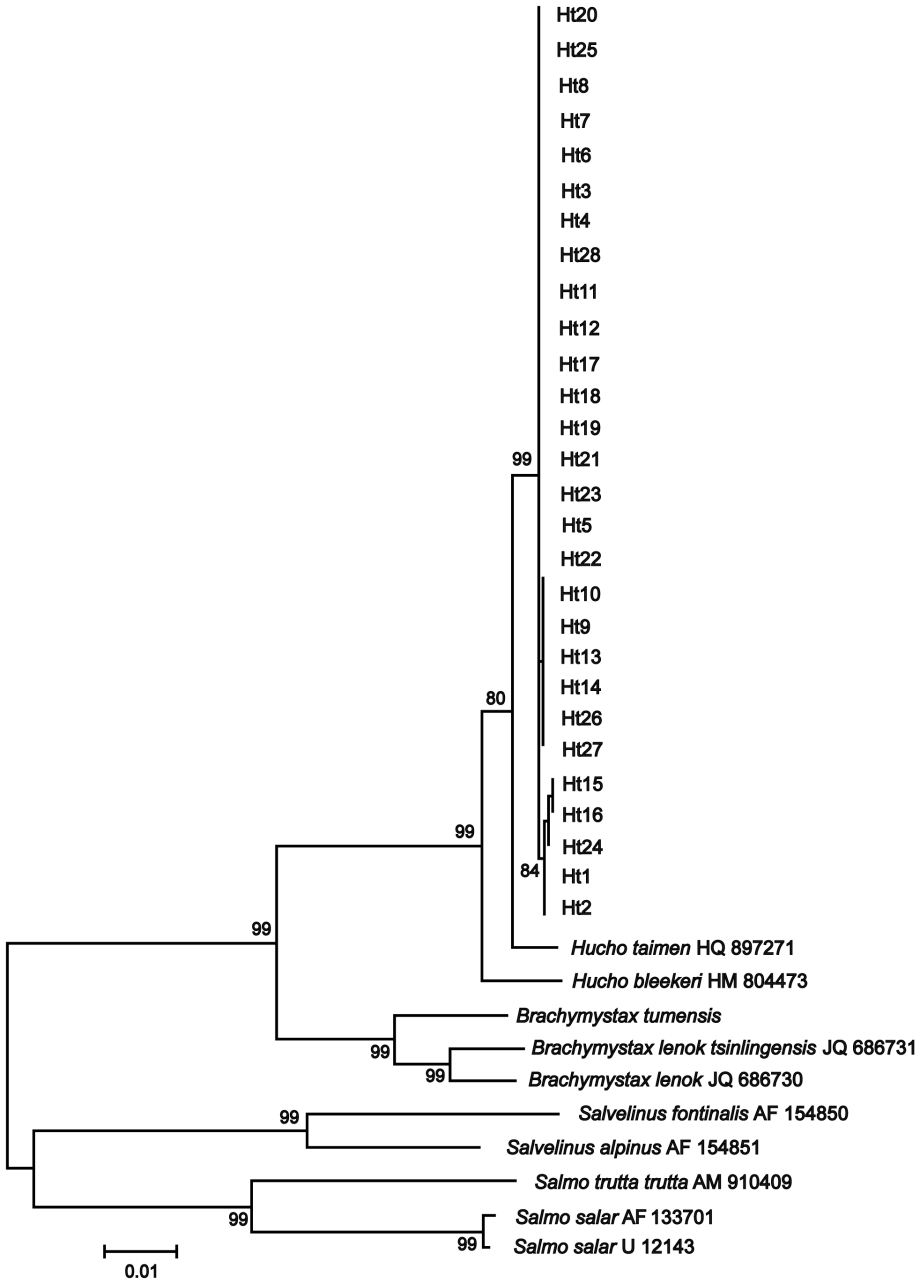
Neighbor-joining tree of the concatenate mitochondrial DNA sequences (8,141 bp) of *Hucho taimen*, based on Tamura-Nei+gamma model of substitution. For tree reconstruction we used the full length of the mitochondrial DNA sequences (8,141 bp), including coding and non-coding regions. Numbers at the nodes are bootstrap percent support values based on 10,000 replications (see Fig. S1 and Table S1 for the collection sites and specimen designation).

A comparison of the data obtained in the present study with the publicly available *H. taimen* mt genome (GenBank HQ897271; the specimen is from Hutou range, Ussuri River, China; thereafter *H. taimen* (Hutou)) revealed significant divergence between them: the total sequence divergence *D*_xy_ = 0.0108±0.0013, is five times higher than the divergence between the two groups of sequences found in our specimens, 0.0021±0.0005. The distance between our specimens and *H. taimen* (Hutou) is below the range of divergence between species, but significantly higher than the divergence between the two groups of sequences in our specimens, and approaches the divergence between two *Hucho* species, *H. taimen* (our data) and *H. bleekeri* (GenBank HM804473) (*D*_xy_ = 0.0194±0.0015). The difference is surprising because the specimens came from close geographical areas in the Ussuri River (Fig. S1). There are approximately 141 miles between the Hutou range (the sampling point of the GenBank specimen) and the Bikin River (the sampling point of specimens 4 and 5 in our study). A phylogram of the *H. taimen* specimens obtained in the present study, along with other salmonids (GenBank data), is displayed in Fig. 2. The tree shows our *H. taimen* specimens forming a single clade; in contrast, the *H. taimen* (Hutou) is distinct with high bootstrap support (99%).

### Sliding Window Analysis

**Figure 3:**
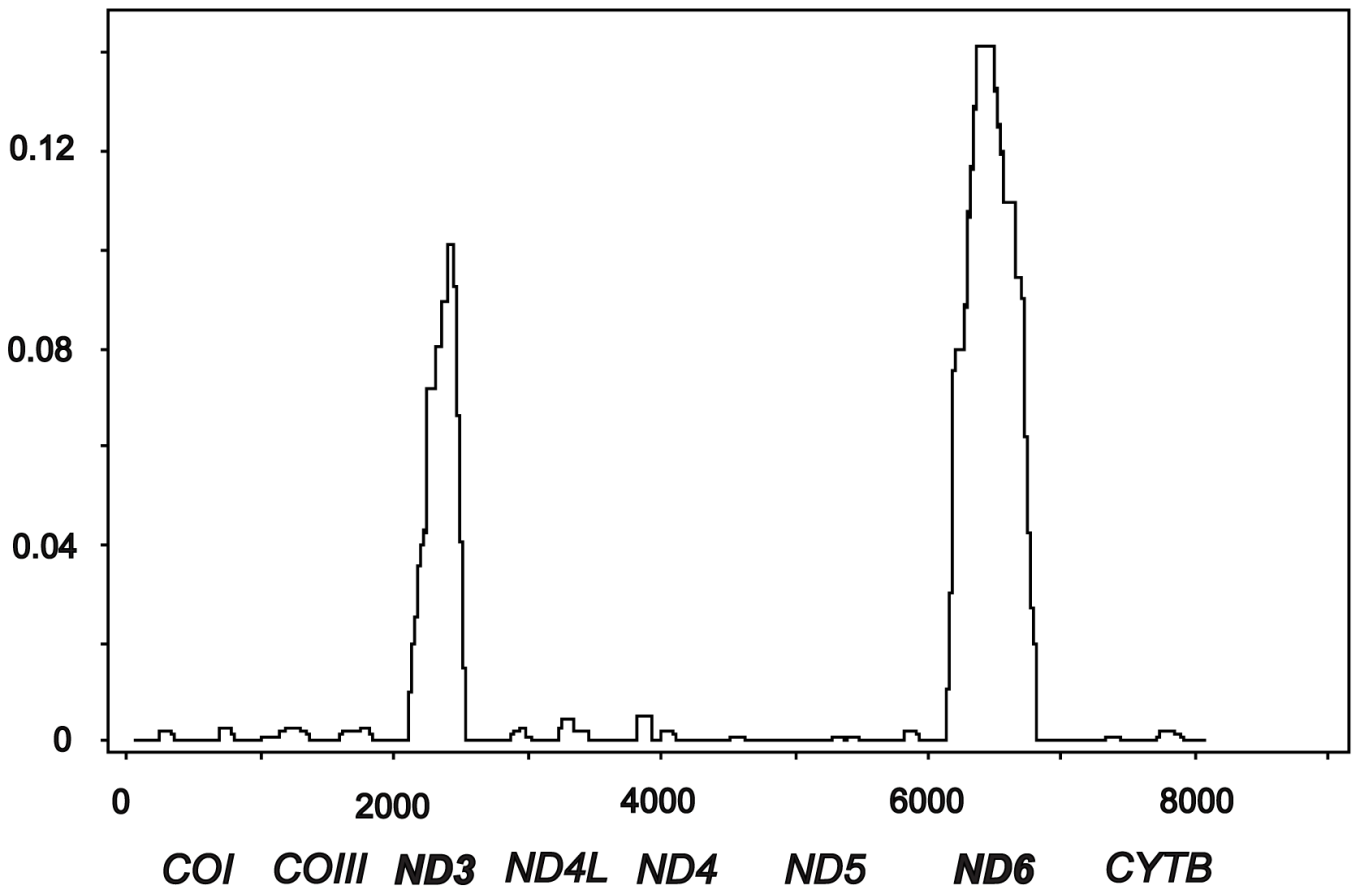
Sliding-window plot of divergence along the concatenate mitochondrial DNA sequences (8,141 bp) between the GenBank *Hucho taimen* mt genome (HQ897271) and the *H. taimen* sequences obtained in the present study. Window sizes are 250 nucleotides with 25-nucleotide increments. The order of the mitochondrial genes is schematically shown at bottom. Note the two significant peaks of divergence centered on two genes, *ND3* and *ND6*.

The distribution of divergence between *H. taimen* specimens obtained in the present work and *H. taimen* (Hutou) was non-uniform, with two compact but highly pronounced peaks centered on two genes, *ND3* and *ND6* (Fig. 3). The same two peaks were detected in a comparison of the full mt genomes of *H. taimen* (Hutou) and *H. bleekeri* (data not shown). Peaks of divergence were not accompanied by increased (or decreased) levels of polymorphism nor by neutrality test statistics (data not shown). The regions of elevated divergence could be considered as “genomic islands of differentiation” (e.g., [67]). The first island of divergence (ID1) consisted of approximately 340 bp and coincided with the *ND3* gene (Fig. 3). The second island of divergence (ID2) comprised approximately 560 bp including the *ND6* gene. The BLAST procedure (limited to the current GenBank submissions) reveals very high similarity (maximal identity = 97–100%) between the ID1 and ID2 regions of *H. taimen* (Hutou) and two subspecies of lenok, *B. lenok* (JQ686730) and *B. lenok tsinlingensis* (JQ675732, JQ686731), respectively. For the full mtDNA fragment investigated the average divergence between *H. taimen* (Hutou) and the two subspecies of lenok was 0.0617±0.0021, but noticeably less, 0.0126±0.0026, for the ID1 plus ID2 regions. Moreover, for ID1 there were not any differences between *H. taimen* (Hutou) and *B. lenok* (JQ686730), whereas for ID2 there were not any differences between *H. taimen* (Hutou) and *B. lenok tsinlingensis* (JQ686731).

**Figure 4:**
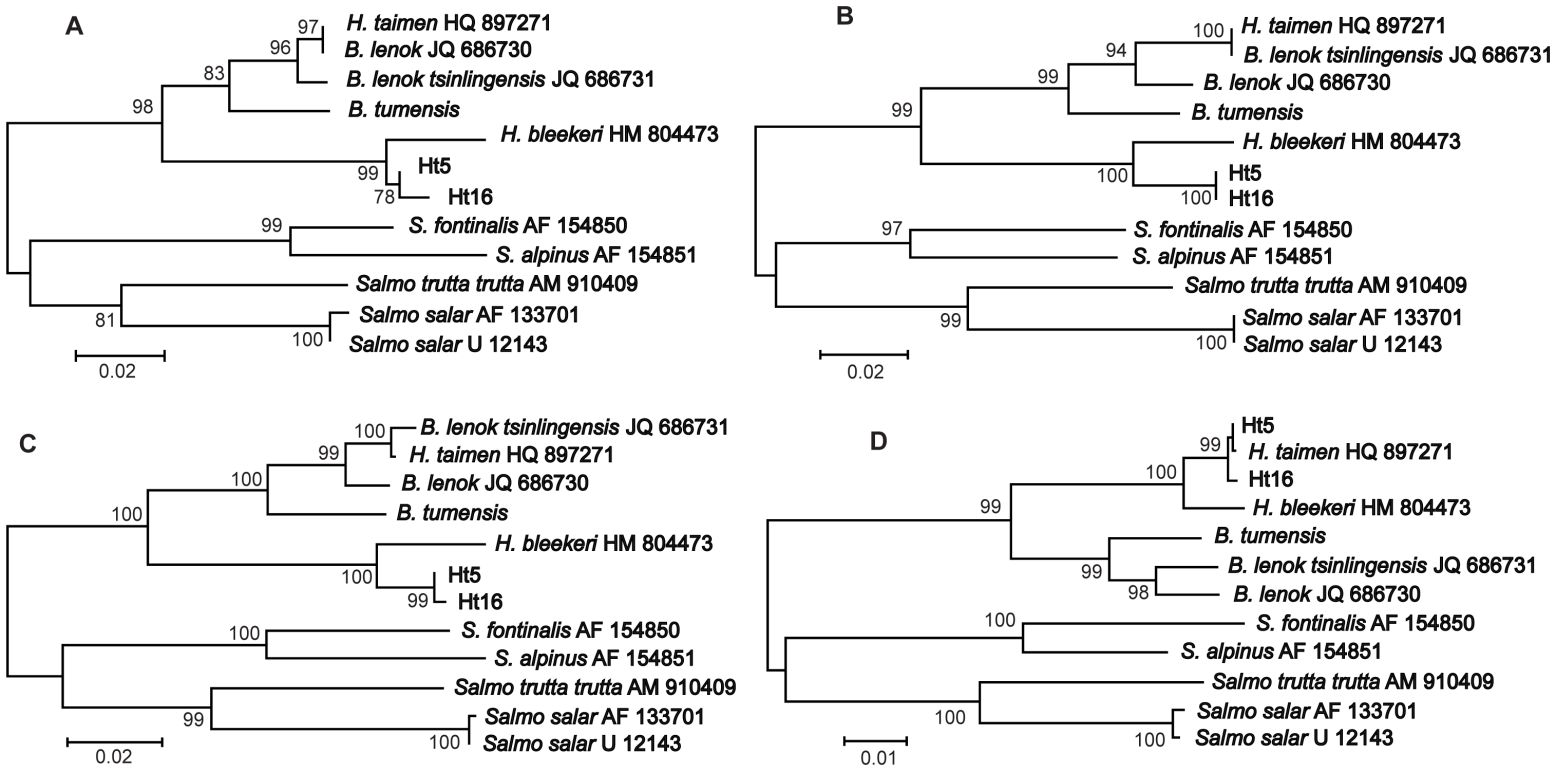
Phylogenetic trees based on different fragments of the mitochondrial DNA sequences: (A) first island of divergence only, (B) second island of divergence only, (C) first plus second islands of divergence, (D) without the two islands of divergence. The tree topologies obtained with Maximum likelihood and Bayesian inference are congruent. Two sequences of *H. taimen*, Ht5 and Ht16, representing haplotype group 1 and 2, are included. The *Salvelinus* and *Salmo* sequences (GenBank accession numbers in Materials and Methods) are used as outgroups. Note the changed position of *H. taimen* from the Hutou range (GenBank HQ897271), depending on the region used for the tree reconstruction.

Thus, there are two regions (ID1 and ID2) within the mtDNA fragment studied which show sharply discordant phylogenetic signals between *Hucho* and *Brachymystax*. As a consequence, the position of *H. taimen* (Hutou) was sharply different, depending on the fragments used for tree reconstruction (Fig. 4). The trees based on ID1 and ID2 separately showed *H. taimen* (Hutou) identical to *B. lenok* (JQ686730) or to *B. lenok tsinlingensis* (JQ686731), respectively (Fig. 4A and Fig. 4B). The tree based on ID1 plus ID2 displayed *H. taimen* (Hutou) between the two lenok species (Fig. 4C). On the tree excluding the ID1 and ID2 regions, *H. taimen* (Hutou) was within the same cluster as the other *H. taimen* specimens (Fig. 4D); the sequence divergence between them was very low, *D*_xy_ = 0.0007±0.0002, three times lower than the divergence between the two groups of sequences detected in our specimens (0.0021±0.0005). Thus, most of the mtDNA fragment of *H. taimen* (Hutou) has obvious similarity to the *H. taimen* sequences obtained in our study, whereas the ID1 and ID2 regions have unexpected similarity to *Brachymystax* subspecies, and could be explained by introgression of mtDNA resulting from hybridization between lenok and taimen. Other salmonids included in this analysis (*S. salmo*, *S. trutta*, *S. fontinalis*, and *S. alpinus*) did not show any visible discordance in the level of divergence between the ID1 and ID2 regions and the rest of the mtDNA (Fig. 4).

### Recombination

**Figure 5:**
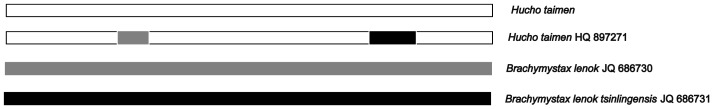
Schematic representation of the recombination events in the mitochondrial DNA of *Hucho taimen* from the Hutou range (GenBank HQ897271). The top parental sequence is from *H. taimen* (our study); the two bottom parental sequences are from *B. lenok* (first recombination event, in grey) and *B. lenok tsinlingensis* (second recombination event, in black).

We suggest that the phylogenetic inconsistencies might reflect hybridization event(s) between *H. taimen* (Hutou) and the two *Brachymystax* subspecies, which might have resulted in interspecific recombination of mitochondrial DNA. The method of Hudson and Kaplan [68] failed to reveal any signal of recombination when we analyzed our specimens. However, two recombination events were detected when we add *H. taimen* (Hutou) in the recombination analysis. We therefore analyzed the mtDNA alignments for evidence of recombination using various recombination detection methods implemented in the program RDP3 [60] (Table 2). All seven methods detected two recombination events in *H. taimen* (Hutou) within the studied region (Fig. 5), with high statistical support (Table 2). For recombination event 1, the major and minor parents were *H. taimen* (our specimens) and *B. lenok*, respectively (breakpoint positions 2116–2561; *P* = 2.984×10^−25^); whereas for the recombination event 2 the major and minor parents were *H. taimen* (our specimens) and *B. lenok tsinlingensis*, respectively (breakpoint positions 6188–6870; *P* = 8.528×10^−42^) (Fig. 5).

**Table 2:** Recombination assessed by seven different methods implemented in RDP3 (see [60]).

Method	Reference	*P* – value 1	*P* – value 2
RDP	[69]	2.884×10^−25^	8.528×10^−42^
GENECONV	[70]	7.343×10^−24^	1.295×10^−39^
BOOTSCAN	[71]–[72]	2.871×10^−25^	6.773×10^−42^
MAXCHI	[73]	2.753×10^−06^	4.089×10^−14^
CHIMAERA	[74]	2.718×10^−06^	2.696×10^−14^
SiScan	[75]	3.358×10^−07^	1.302×10^−15^
3Seq	[76]	2.903×10^−05^	3.671×10^−32^

Note. – *P* – value 1 and *P* – value 2 are the average *P* – values for the recombination even 1 and 2, respectively. For the recombination analysis we used the full mtDNA segment sequenced (8,141 bp). The analysis included 38 sequences: 28 *H. taimen* sequences (our data), *H. taimen* (Hutou), *H. bleekeri*, *B. lenok*, *B. lenok tsinlingensis* (two sequences), *B. tumensis*, *S. salar*, *S. trutta*, *S. fontinalis*, and *S. alpinus*. For GenBank accession numbers see Materials and Methods.

Thus, the recombination analysis confirms that the mtDNA of *H. taimen* (Hutou) is a recombinant product between the mtDNA of *H. taimen* and each of two lenok subspecies, *B. lenok* and *B. lenok tsinlingensis*. The location of the recombinant fragments corresponds to the ID1 and ID2 regions described above (see Figs. 3 and 5). Interestingly, recombination was not detected in our *H. taimen* specimens, which, together with full identity of the introgressed fragments between *H. taimen* (Hutou) and the two *Brachymystax* subspecies, suggests contemporary local introgression. These results are relevant concerning the DNA barcoding for fishes (and possibly other organisms where introgression may have happened). Interspecific mtDNA recombination such as detected in the *ND3* and *ND6* genes of *H. taimen* (the present data) and *ND1* gene of *Salmo* [77] and *Salvelinus* [78] (see Discussion), may importantly limit the usefulness of mtDNA barcoding for species identification.

## Discussion

Introgression is frequent in fish but most observations are based on indirect evidence.

There are multiple examples of introgression in salmonid (as well as in many others) fishes (review in [38], [79]–[80]). There are also multiple evidence of mtDNA recombination in plants, fungi, and animals, including humans (review in [81]–[82]). However, instances of interspecific mtDNA recombination have been rarely detected in hybridizing conifers [83], salmonids, *Salmo* and *Salvelinus* [77]–[78], and primates [84], which could probably be explained by fact that in most cases the data obtained involve relatively short DNA fragments or indirect genetic markers like allozymes, PCR-RFLP or microsatellites. The indirect approaches, albeit may yield correct conclusions about introgression, do not elucidate the precise architecture of the introgression events. The use of full (or big segments of) mtDNA genome allows to reveal and analyze introgression more precisely.

### Localization and Size of the Introgressed Fragments

Using the 8,141 bp fragment of mt genome we detected two recombinant fragments, which could be accounted for by introgression of mtDNA due to hybridization between each of two subspecies of lenok, *B. lenok* and *B. lenok tsinlengensis*, and Siberian taimen *H. taimen*. The introgression is limited to two relatively small regions (around 0.9 kb) including two genes, *ND3* and *ND6*. Our results are consistent with recent data from a genome scan of the bivalve mollusk *Mytilus galloprovincialis* [85]. Using AFLP markers Gosset and Bierne [85] detected a small fraction (around 2%) of outlier loci with high *F*_ST_ and proved that the high divergence was due to differential introgression of alleles from the sister-hybridizing species *Mytilus edulis*. Consequently, introgression would seem possible and frequent enough to account for “genomic islands of differentiation” [67], which are often interpreted as a result of spatially heterogeneous selection [86].

### The *ND6* Gene is Usually Excluded from Phylogenetic Analysis

The *ND6* mtDNA gene is usually excluded from phylogenetic analyses of vertebrates [87]–[88]. The reasons are diverse: 1) *ND6* is the only protein-coding gene encoded on the light strand and consequently has quite different evolutionary properties from those of the other 12 protein genes, making it an inappropriate mix with model-based methods; 2) the presence of many indels; 3) heterogeneous base composition; 4) consistently poor phylogenetic performance; and finally 5) difficulties with Kappa (transition-transversion ratio) estimation. Consequently, *ND6* is usually ignored (references include a large majority of papers in the field of “mitogenomics” published during the last twelve years). The *ND6* gene was also excluded by Wang et al. [48] and Si et al. [49] investigating mt genomes of taimen *H. bleekeri* and lenok subspecies *B. lenok* and *B. lenok tsinlingensis*. The mt genome of *H. taimen* (Hutou) was not analyzed using a phylogenetic approach [47]. Our results show that *ND6* (along with *ND3*) provides clear evidence of gene flow between *Brachymystax* subspecies and *H. taimen* and could be used as a valuable marker to detect intergeneric hybridization between them. Similar patterns could occur in other vertebrates, suggesting the *ND6* gene should be included in at least preliminary phylogenetic analysis. Otherwise an interesting feature of fish mitochondrial genome evolution with important practical consequences might be missed.

### Is Introgression due to Natural or Artificial Hybridization?

We detected full identity in two short introgressed mtDNA fragments between *H. taimen* (Hutou) and two lenok subspecies, *B. lenok* and *B. lenok tsinlingensis*. Full identity suggests contemporary introgression in both cases (*B. lenok* - *H. taimen* and *B. lenok tsinlingensis* - *H. taimen*). A remaining question concerns the mechanism of hybridization. Natural hybrids between *H. taimen* and *B. lenok* have been registered in the areas of sympatry [32]–[34]. Consequently, the introgressed mtDNA fragments in *H. taimen* (Hutou) could be explained by natural hybridization events. However, the involvement of *B. lenok tsinlingensis* can hardly be explained by natural hybridization, because this subspecies is endemic to the Huanghe River basin [3], [49], which is disjunct from the Amur River basin at the present time. Moreover, this subspecies is rare; it has been listed in the China Red Data Book of Endangered Animals [6]. It might be suggested that the rate of mtDNA evolution is extremely low in *H. taimen* and that introgressed fragment is a “frozen” relict from Pleistocene time, when the Huanghe and Amur River basins were connected [89]–[90]. We consider this alternative hypothesis (low rate of mtDNA evolution in *H. taimen*) highly improbable, especially taking into account the data of Xia et al. [91] on the phylogeographic structure of *B. lenok* (which is close to *B. lenok tsinlingensis*). Using two mtDNA fragments (control region, 835 bp and *CYTB*, 1,069 bp) they detected strong geographic differentiation among *B. lenok* populations. Particularly, they found significant difference between lenok inhabiting the Huanghe and Amur River basins, with the average sequence divergence 1.93%. Consequently, the full identity of the introgressed fragments identified in *H. taimen* (Hutou) could not likely be a product of natural hybridization, but rather it would likely be due to artificial hybridization experiments under laboratory conditions. The absence of introgression in the *H. taimen* specimens collected from close localities in the Ussuri River (but in Russian territory) further supports this hypothesis. Indeed, a technique for artificial reproduction between *H. taimen* and *B. lenok* has been developed [25]. Artificial intergeneric hybrids have been reported between *H. taimen* and *B. lenok* [25]–[27] but not between *H. taimen* and *B. lenok tsinlingensis*. The fact that the hybrid *H. taimen* individual was captured in a natural environment (Hutou range, Ussuri River, Heilongjiang Province) [47] suggests the possibility of hybrid individuals escaping from fish rearing farms.

### The Negative Consequences of Human-mediated Hybridization

Interspecific hybridization has been described for many fish species (review in [79], [92]–[94]). Hybrid speciation is widespread in plant and animal evolution (e.g., [95]–[97] and references therein) and mosaic genomes due to hybrid speciation are common in microorganisms, plants and animals [98]. Natural introgressive hybridization does not require special attention in terms of conservation and can indeed play a positive role as a source of variability [99]–[100]. However, human-mediated hybridization represents a major threat to aquatic biodiversity worldwide, leading to the erosion of local genetic diversity with loss of adaptive potential and negative fitness consequences (review in [92], [101]–[103]). Captive propagation can help reduce the risk of short-term extinction, but ecological and genetic interactions between wild and hatchery fishes may have negative consequences for wild populations, including loss of genetic variability, loss of adaptations, and change of population make-up and structure (review in [104]–[106]). Thus, artificial breeding, hybridization, and captive propagation cannot substitute for conserving the species in the wild.

There have been some important successes for *H. taimen* in aquaculture practice and propagation [22]–[29], but these activities should proceed with great caution and in conjunction with a full assessment of risks and benefits. Human-induced genetic erosion of *H. taimen* populations could be provoked by releases of farm-reared *H. taimen* and *H. taimen* – *B. lenok* hybrids; genetic and ecological risks associated with the releases should not be neglected in management and policy. The significant threat of human-mediated hybridization demands a responsible breeding program and management, in order to prevent potential spread of hybrid fishes that can jeopardize the resilience of locally adapted gene pools of native *H. taimen* populations. Genetic monitoring offers an important and reliable approach to discriminate hybrid individuals and to assess the threat from hybridization to the genetic integrity of *H. taimen* natural populations.

The mtDNA data clearly show hybridization and introgression between *H. taimen* and two lenok subspecies, *B. lenok* and *B. lenok tsinlingensis*. Further evidence from biparentally inherited nuclear DNA will be required to critically evaluate the revealed patterns, in particular to determine what fraction of the extant *H. taimen* genome has been impacted by gene flow between *H. taimen* and lenok subspecies.

## Supporting Information

Figure S1 **Sampling locations of** ***Hucho taimen*** **.** Arabic numerals (1–6) correspond to sample sites. The sample site from China (Hutou range, the Ussuri River) is marked by an asterisk. See Table S2 for river names, basins, sample sizes, and coordinates.
(TIF)

Table S1 ***Hucho taimen*** **(Ht) specimens and collection sites.**
(DOCX)

Table S2 **River names, basins, sample sizes, and coordinates for the** ***Hucho taimen*** **specimens used in this study.**
(DOCX)

Table S3 **Primers used in the present study.**
(DOCX)

Table S4 **Region of the** ***Hucho taimen*** **mitochondrial genome studied in the present work, with corresponding coordinates in the mitochondrial genomes of** ***H. taimen*** **,** ***H. bleekeri*** **, and** ***Brachymystax lenok tsinlingensis*** **.**
(DOCX)

Table S5 **Best-fit models of mitochondrial protein-coding genes evolution in** ***Hucho taimen*** **.**
(DOCX)
