# Supplementary figures and images for: Mitochondrial DNA Variation and Introgression in Siberian Taimen Hucho taimen
Source: PLoS One. 2013 Aug 12;8(8):e71147. doi: 10.1371/journal.pone.0071147 (PMC3741329; doi:10.1371/journal.pone.0071147)

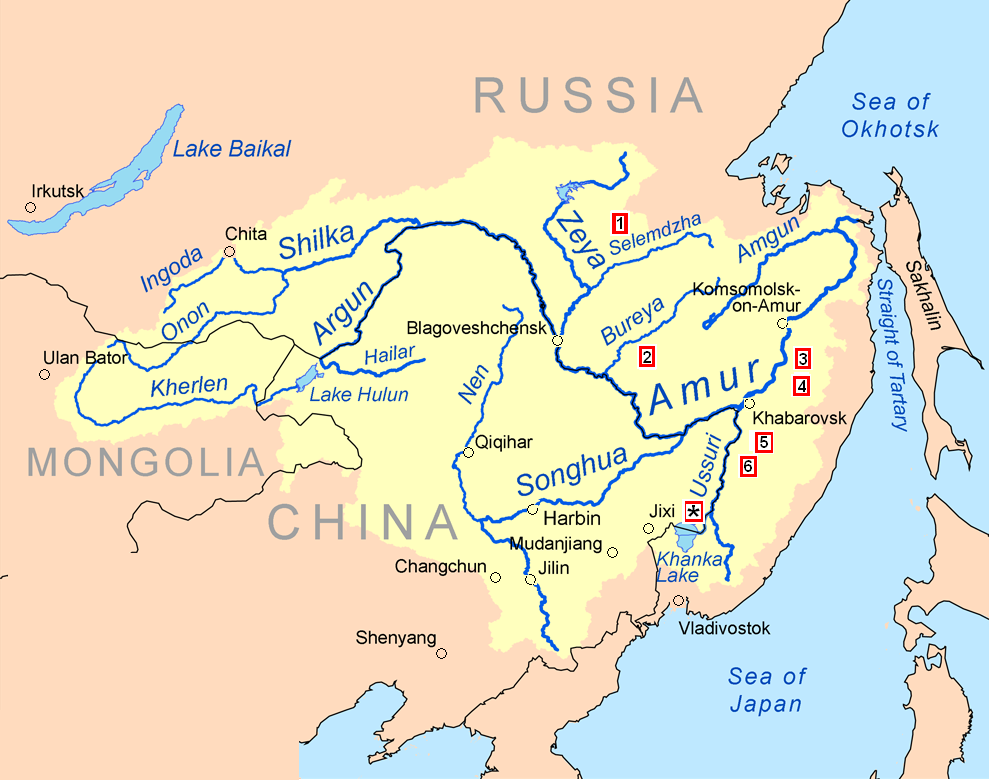

Supplement: Figure S1 — Sampling locations of Hucho taimen . Arabic numerals (1–6) correspond to sample sites. The sample site from China (Hutou range, the Ussuri River) is marked by an asterisk. See Table S2 for river names, basins, sample sizes, and coordinates. (TIF) [file pone.0071147.s001.tif]
