# Supplementary material for: Mitochondrial DNA Variation and Introgression in Siberian Taimen Hucho taimen
Source: PLoS One. 2013 Aug 12;8(8):e71147. doi: 10.1371/journal.pone.0071147 (PMC3741329; doi:10.1371/journal.pone.0071147)
Supplement: Table S1 — Hucho taimen (Ht) specimens and collection sites. (DOCX) [file pone.0071147.s002.docx]

Table S1. *Hucho taimen* (Ht) specimens and collection sites

| Fish No. | Locality | Region | Comments |
| --- | --- | --- | --- |
| Siberian taimen *Hucho taimen* (Pallas, 1773) | | | |
| Ht1 | Nora | AR | ♀ |
| Ht2 | Nora | AR | ♂ |
| Ht3 | Nora | AR | na |
| Ht4 | Bikin | PT | im |
| Ht5 | Bikin | PT | im |
| Ht6 | Manoma | KhT | na |
| Ht7 | Anyuy | KhT | ♀II |
| Ht8 | Sutara | JAR | ♂I |
| Ht9 | Khor | KhT | ♂I |
| Ht10 | Khor | KhT | ♀I |
| Ht11 | Khor | KhT | na |
| Ht12 | Khor | KhT | ♂I |
| Ht13 | Khor | KhT | ♀II |
| Ht14 | Khor | KhT | ♀ |
| Ht15 | Khor | KhT | na |
| Ht16 | Khor | KhT | ♀ |
| Ht17 | Khor | KhT | na |
| Ht18 | Khor | KhT | mat ♂III |
| Ht19 | Khor | KhT | ♀II |
| Ht20 | Khor | KhT | ♂I |
| Ht21 | Khor | KhT | ♀I |
| Ht22 | Khor | KhT | ♀ |
| Ht23 | Khor | KhT | ♂ |
| Ht24 | Anyuy | KhT | ♂ |
| Ht25 | Anyuy | KhT | na |
| Ht26 | Anyuy | KhT | ♀ |
| Ht27 | Anyuy | KhT | na |
| Ht28 | Anyuy | KhT | na |
| Blunt-snouted lenok *Brachymystax tumensis* Mori, 1930 | | | |
| 1 | Bikin | PT | mat |

AR – Amur Region; PT – Primorye Territory; KhT – Khabarovsk Territory; JAR – Jewish Autonomous Region; im – sexually immature individual; mat – mature individual; Roman numeral close to sex designation denotes the stage of sexual maturity; na – information is not available.
