# Supplementary material for: Mitochondrial DNA Variation and Introgression in Siberian Taimen Hucho taimen
Source: PLoS One. 2013 Aug 12;8(8):e71147. doi: 10.1371/journal.pone.0071147 (PMC3741329; doi:10.1371/journal.pone.0071147)
Supplement: Table S2 — River names, basins, sample sizes, and coordinates for the Hucho taimen specimens used in this study. (DOCX) [file pone.0071147.s003.docx]

Table S2. River names, basins, sample sizes, and coordinates for the *Hucho taimen* specimens used in this study. (See also Table S1 and Fig. S1)

| Sampling site | River | Basin | Sample size | Coordinates |
| --- | --- | --- | --- | --- |
| 1 | Nora | Zeya | 3 | 53° 8'17.73" 130° 3'33.69" |
| 2 | Sutara | Bira (middle Amur region) | 1 | 48°54'9.24" 131° 30'49.09" |
| 3 | Manoma | Anuyu | 1 | 49°28'21.80" 137° 24'58.50" |
| 4 | Anyuy | Lower Amur region | 6 | 49°13'29.78" 137° 8'14.66" |
| 5 | Khor | Ussuri | 15 | 47°30'24.94" 136° 1'24.84" |
| 6 | Bikin | Ussuri | 2 | 46°36'36.54" 136°32'58.94" |
