# Supplementary material for: Mitochondrial DNA Variation and Introgression in Siberian Taimen Hucho taimen
Source: PLoS One. 2013 Aug 12;8(8):e71147. doi: 10.1371/journal.pone.0071147 (PMC3741329; doi:10.1371/journal.pone.0071147)
Supplement: Table S3 — Primers used in the present study. (DOCX) [file pone.0071147.s004.docx]

Table S3. Primers used in the present study

------------------------------------------------------------

Primer[1]

Forward:ACCTCTTGCCTGAAGGAACTC Length:21 Tm:57.6099 GC%:52.381

Reverse:CTGTGGCGACAAAGAAAGTG Length:20 Tm:56.5233 GC%:50.0

Primer[2]

Forward:AGTCCTTCTAGCATCTGGTGTC Length:22 Tm:55.2704 GC%:50.0

Reverse:AAAGGCTGAGGTAAAGCTGAAG Length:22 Tm:57.928 GC%:45.4545

Primer[3]

Forward:GTTAGTCCAAAACAAGACCCTTG Length:23 Tm:57.8027 GC%:43.4783

Reverse:CTAAGAGGCAGGCAGCTCATC Length:21 Tm:59.368 GC%:57.1429

Primer[4]

Forward:CCAACGAACTTACATCTCCCTTTTG Length:25 Tm:63.5609 GC%:44.0

Reverse:AGGTAGGGCGAGATTGGCTAAAC Length:23 Tm:63.1981 GC%:52.1739

Primer[5]

Forward:ACACGGTCTTGCCTCCTCAG Length:20 Tm:59.7707 GC%:60.0

Reverse:TGAAATATGTTGTTGGCGGTT Length:21 Tm:58.269 GC%:38.0952

Primer[6]

Forward:GACCAAGGCACCGAAACAATTG Length:22 Tm:64.1502 GC%:50.0

Reverse:CTGCTAAAAATGGAGTGCCAGTAAG Length:25 Tm:62.0455 GC%:44.0

Primer[7]

Forward:TGCTCCGGCTCAATTATTCATAG Length:23 Tm:61.4475 GC%:43.4783

Reverse:TTAACACGGGGGTTGAGTCG Length:20 Tm:60.6793 GC%:55.0

Primer[8]

Forward:ATCGCACTAGAACTTGCATCAC Length:22 Tm:57.2491 GC%:45.4545

Reverse:TTCTTGTAGTTGAATAACAACGG Length:23 Tm:54.878 GC%:34.7826

Primer[9]

Forward:CACAAGCCCTAAAACCAACCC Length:21 Tm:61.1839 GC%:52.381

Reverse:TTGGCGGGTGTAAAATTGTCTG Length:22 Tm:62.5553 GC%:45.4545

Primer[10]

Forward:ATTTTTCGCCTTCCACTTCCTATTC Length:25 Tm:63.7865 GC%:40.0

Reverse:AGTTGGTGGTTATCCGTGTTTTG Length:23 Tm:60.8641 GC%:43.4783

Primer[11]

Forward:AAACCCTCCCTAGTGCTCAGAG Length:22 Tm:59.4708 GC%:54.5455

Reverse:CTGTTGAGACTTCCTGGTTTAGGG Length:24 Tm:61.4237 GC%:50.0

-------------------------------------------------------------

The primers are designed with the program MitoPrimerV1 [1].

**References**

1. Yang C-H, Chang H-W, Ho C-H, Chou Y-C, Chuang L-Y (2011) Conserved PCR primer set designing for closely-related species to complete mitochondrial genome sequencing using a sliding window-based PSO algorithm. PLoS ONE 6(3): e17729.
