# Supplementary material for: Mitochondrial DNA Variation and Introgression in Siberian Taimen Hucho taimen
Source: PLoS One. 2013 Aug 12;8(8):e71147. doi: 10.1371/journal.pone.0071147 (PMC3741329; doi:10.1371/journal.pone.0071147)
Supplement: Table S4 — Region of the Hucho taimen mitochondrial genome studied in the present work, with corresponding coordinates in the mitochondrial genomes of H. taimen , H. bleekeri , and Brachymystax lenok tsinlingensis . (DOCX) [file pone.0071147.s005.docx]

Table S4. Region of the *Hucho taimen* mitochondrial genome studied in the present work, with corresponding coordinates in the mitochondrial genomes of *H. taimen*, *H. bleekeri*, and *Brachymystax lenok tsinlingensis*

| *H. taimen* [1] | *H. bleekeri* [2] | *B. lenok. tsinlingensis* [3] | Gene | Present work | S | Comments |
| --- | --- | --- | --- | --- | --- | --- |
| 6663 – 8195 | 6827 – 8360 | 6499 – 8031 | *COI* | 1 – 1533 | 6 | 15 bp (5’) are not sequenced |
| 8196 – 10187 | 8361 – 10353 | 8032 – 10023 | tRNA-Ser, tRNA-Asp, *COII*, tRNA-Lys, *ATP8*, *ATP6* |  |  | Not sequenced region |
| 10188 – 10750 | 10354 – 10914 | 10024 – 10587 | *COIII* | 1534 – 2096 | 3 | 222 bp (5’) are not sequenced |
| 10751 – 10820 | 10915 – 10984 | 10587 – 10656 | tRNA-Gly | 2097 – 2166 | 0 |  |
| 10821 – 11169 | 10985 – 11333 | 10657 – 11005 | *ND3* | 2167 – 2515 | 2 | First divergence peak (centered on ≈ 2400) |
| 11170 – 11239 | 11334 – 11403 | 11006 – 11075 | tRNA-Arg | 2516 – 2585 | 0 |  |
| 11240 – 11536 | 11404 – 11700 | 11076 – 11372 | *ND4L* | 2586 – 2882 | 0 |  |
| 11530 – 12910 | 11694 – 13074 | 11366 – 12746 | *ND4* | 2876 – 4256 | 7 | 7 bp overlapping with *ND4L* (5’) |
| 12911 – 12979 | 13075 – 13143 | 12747 – 12815 | tRNA-His | 4257 – 4325 | 0 |  |
| 12980 – 13048 | 13144 – 13212 | 12816 – 12884 | tRNA-Ser | 4326 – 4394 | 0 |  |
| 13049 | 13213 | 12885 |  | 4395 | 0 | 1-bp (C) spacer |
| 13050 – 13122 | 13214 – 13286 | 12886 – 12958 | tRNA-Leu | 4396 – 4468 | 0 |  |
| 13123 – 14961 | 13287 – 15125 | 12959 – 14797 | *ND5* | 4469 – 6307 | 5 | 4 bp overlapping with *ND6* (5’) |
| 14958 – 15479 | 15122 – 15643 | 14794 – 15315 | *ND6* | 6304 – 6825 | 3 | Second divergence peak (centered on ≈ 6500) |
| 15480 – 15548 | 15644 – 15712 | 15316 – 15384 | tRNA-Glu | 6826 – 6894 | 0 |  |
| 15549 – 15551 | 15713 – 15715 | 15385 – 15387 |  | 6895 – 6897 | 0 | 3-bp (CTA) spacer |
| 15552 – 16692 | 15716 – 16856 | 15388 – 16528 | *CYTB* | 6898 – 8038 | 6 |  |
| 16693 – 16764 | 16857 – 16928 | 16529 – 16600 | tRNA-Thr | 8039 – 8110 | 0 |  |
| 16764 – 16795 | 16928 – 16959 | 16600 – 16631 | tRNA-Pro | 8110 – 8141 | 0 | 37 bp (3’) are not sequenced |

S, polymorphic sites.

**References**

1. Wang Y, Zhang X-Y, Yang S-Y, Song Z-B (2011a) The complete mitochondrial genome of the taimen, *Hucho taimen*, and its unusual features in the control region. Mitochondrial DNA 22:111–119.

2. Wang Y, Guo R, Li H, Zhang X-Y, Du J, Song Z-B (2011b) The complete mitochondrial genome of the Sichuan taimen (*Hucho bleekeri*): repetitive sequences in the control region and phylogenetic implications for salmonidae. Marine genomics 4:221-228.

3. Si S-J, Wang Y, Xu G-F, Yang S-Y, Mou Z-B, Song Z-B (2012) Complete mitochondrial genomes of two lenoks, *Brachymystax lenok* and *Brachymystax lenok tsinlingensis*. Mitochondrial DNA 23: 338-340.
