# Supplementary material for: Mitochondrial DNA Variation and Introgression in Siberian Taimen Hucho taimen
Source: PLoS One. 2013 Aug 12;8(8):e71147. doi: 10.1371/journal.pone.0071147 (PMC3741329; doi:10.1371/journal.pone.0071147)
Supplement: Table S5 — Best-fit models of mitochondrial protein-coding genes evolution in Hucho taimen . (DOCX) [file pone.0071147.s006.docx]

Table S5. Best-fit models of mitochondrial protein-coding genes evolution in *Hucho taimen*

| Genes | Sequence length (bp) | Best fit model | BIC | AIC | *lnL* |
| --- | --- | --- | --- | --- | --- |
| *COI* | 1530 | TN93+G | 9822.236 | 9093.735 | -4465.756 |
| *COIII* | 561 | HKY+G | 3977.719 | 3338.851 | -1589.128 |
| *ND3* | 348 | K2+G | 3026.969 | 2449.145 | -1147.127 |
| *ND4L* | 294 | K2+I | 2364.204 | 1799.529 | -822.869 |
| *ND4* | 1380 | HKY+G | 3977.719 | 3338.851 | -1589.128 |
| *ND5* | 1836 | TN93+G | 12314.106 | 11570.844 | -5704.329 |
| *ND6* | 519 | HKY+G | 4217.140 | 3584.546 | -1711.951 |
| *CYTB* | 1140 | TN93+G | 8002.146 | 7297.556 | -3567.628 |
| Full length | 7608 | TN93+G | 49332.434 | 48473.847 | -24155.901 |

TN93: Tamura-Nei [1]; HKY: Hasegawa-Kishino-Yano [2]; K2: Kimura 2-parameter [3]. Models with the lowest Bayesian Information Criterion (BIC) scores [4] are considered to describe best the substitution pattern. For each model, Akaike Information Criterion (AIC) [5] and maximum likelihood value (*lnL*) [6] are also presented. G and I refer to the gamma shape parameter and estimated fraction of invariant sites.

**References**

1. Tamura K, Nei M (1993) Estimation of the number of nucleotide substitutions in the control region of mitochondrial DNA in humans and chimpanzees. Mol Biol Evol 10: 512–526.

2. Hasegawa M, Kishino H, Yano T (1985) Dating of human–ape splitting by a molecular clock of mitochondrial DNA. J Mol Evol 22: 160–174.

3. Kimura M (1980) A simple method for estimating evolutionary rate of base substitutions through comparative studies of nucleotide sequences. J Mol Evol 16: 111-120.

4. Schwarz GE (1978) Estimating the dimension of a model. Ann Stat 6: 461–464.

5. Akaike H (1974) A new look at the statistical model identification. IEEE Trans Automat Contr 19: 716–723.

6. Nei M, Kumar S (2000). Molecular Evolution and Phylogenetics. New York: Oxford University Press. 333 p.
